# Acclimation and Blood Sampling: Effects on Stress Markers in C57Bl/6J Mice

**DOI:** 10.3390/ani13182816

**Published:** 2023-09-05

**Authors:** Nerea Marin, Amparo Moragon, Domingo Gil, Francisco Garcia-Garcia, Viviana Bisbal

**Affiliations:** 1 Animal Facility IISLAFE, Hospital La Fe Research Institute, 46026 Valencia, Spain; nerea_marin@iislafe.es; 2 Animal Facility CIPF, Prince Felipe Research Center, 46012 Valencia, Spain; amoragon@cipf.es (A.M.); dgil@cipf.es (D.G.); 3 Bioinformatics & Biostatistics Unit CIPF, Prince Felipe Research Center, 46012 Valencia, Spain; fgarcia@cipf.es

**Keywords:** blood sampling, mice, stress, glucose refinement, 3R, acclimation, animal welfare

## Abstract

**Simple Summary:**

Blood sampling from laboratory animals is a routine procedure in biomedical research, and husbandry is known to affect the quality of animal models. The effect of handling required by these techniques can have a major impact on the condition and responses of experimental animals. Comparing three common methods of blood extraction in mice, i.e., saphenous vein phlebotomy, caudal vein phlebotomy and tail cut blood collection, together with the effect of acclimation, we study which technique is less stressful for the animal. Our results suggest that saphenous vein phlebotomy causes less stress even when acclimation is not performed.

**Abstract:**

Blood sampling in rodents is common practice in scientific studies. Some of the refined methods widely used are the puncture of the saphenous vein or tail vein, or even tail docking. The handling needs of these different blood sampling methods are different and can directly affect stress, increasing the variability of the study. Moreover, there is less aversion and stress if the animal is accustomed to the environment, handling and technique. Therefore, our study aimed to assess the influence of these three blood sampling techniques (saphenous puncture, tail vein puncture and tail vein docking) and the use of previous acclimation on different indicators of animal stress, assessing blood glucose concentrations and faecal corticosterone metabolites (FCMs). Twenty-four young adult male and female C57Bl6/J mice were divided in three groups by sampling method: tail docking (TD), saphenous vein puncture (SV) and caudal vein puncture (CV) groups. All mice were studied with and without acclimation, which was performed during 9 consecutive days. The results showed that both males and females present very similar responses to the different handling and sampling methods without significant differences. Nevertheless, acclimation in all sampling methods decreased glucose and FCM levels significantly. The method that obtained the lowest glucose and FCM levels with significance was saphenous vein puncture. Therefore, we can say that it causes less stress when performing prior acclimation, even when this involves greater handling of the animal. Our results contribute to refinement within the 3R concept and could serve researchers to programme and select a good handling technique and a welfare-friendly blood sampling method for their experiments.

## 1. Introduction

“Good welfare equals good science” was demonstrated by Trevor Poole in 1997 [1], and, according to the principles of replacement, reduction and refinement, adverse effects such as pain, fear and distress should be avoided or minimised [2]. In addition, the refinement of techniques is one of the most important principles in laboratory animal science and must be considered an ethical and legal requirement. Blood sampling is a common procedure in biomedical research and could be a source of stress that can affect the variability of results and compromise animal welfare. Different scientific organisations have published recommendations and guidelines for commonly used blood sampling techniques in laboratory mice [3,4,5], but the techniques differ in their degree of invasiveness and in the handling duration needed [6,7], which might provoke different grades of distress in the animals. In this sense, any pain or distress has to be an objective to avoid in all experiments, and the optimal method needs to be used for collecting blood to accomplish minimal stress according to principles of replacement, reduction and refinement [8].

The literature describes different methods of blood sampling with more or less restraint techniques in mice. Hurst and West [9] showed that the handling method itself could be critical and can induce fear and anxiety responses to human contact. They found that picking mice up in a handling tunnel or cupping them in the open hand leads to substantial voluntary interaction with the handler and reduces stress and anxiety. Some blood sampling methods used in mice require intense handling (vena facialis puncture, retrobulbar sinus puncture, sublingual vein puncture, saphenous phlebotomy, etc.), and this makes them probably more stressful than those methods needing less handling. Meyer demonstrated that a single blood collection from the vena facialis, retrobulbar sinus or tail vessel led to an acute increase in plasma corticosterone levels, with a strong response when sampling from the facial vein and retrobulbar sinus [10]. It has been described that mice respond negatively when the base of the tail is picked up and do not readily habituate to this widely used method. Furthermore, the use of non-aversive tunnels or cupping methods has shown a reduction in plasma corticosterone and glucose levels [11] and reduced pain grimace scores when compared with tail-handled mice [12]. Some studies have shown the influence of handling on animal welfare and how acclimation reduces stress in mice [13,14,15,16].

Several studies compare the quality of blood samples obtained by different blood sampling methods [4,11,17,18]. Facial vein phlebotomy is a common blood sampling method in mice due to its simplicity and good quality of blood but requires high restraint. It allows a maximum allowable sample with minimal trauma [19]. Tail vein sampling is a quick and simple method that can be practised with a bit of restraint or without restraint by senior practitioners but requires some dilatation of blood vessels and does not allow maximum volumes to be obtained. Lateral saphenous vein puncture is a refined method of blood sampling, which is relatively quick but requires a moderate-to-high restraint technique [18,20].

The literature describes different methods of blood sampling with more or less restraint techniques in mice. Manipulation and restraint can have a great impact on animal welfare and could result in different responses of laboratory animals. In fact, handling stress is often pointed out as a potential source of unexplained variability of results [21]. This is due to the influence of management on both behaviour and physiology of animals [22,23], and not considering this might lead to an increase in the number of animals required for experiments. Animals in captivity are very sensitive to interaction with humans, so handling is habitually an unavoidable and variable procedure and animals have to be familiarised to allow them to learn that these interactions are not harmful [24].

Blood sampling remains the most widely used method for the assessment of hypothalamic–pituitary–adrenal (HPA) axis activity, and there are currently no appropriate alternative methods to assess acute changes [4,11,25]. It is essential to minimise the stress associated with these techniques and refine to the maximum the techniques used.

Plasma glucose levels are one of the blood parameters used as indicators of stress in rodents [21,26], since stress-mediated corticosterone production leads to gluconeogenesis and inhibition of insulin secretion [22]. These studies compare the glucose levels associated with the blood sampling technique but do not try to minimise these with prior acclimation of the animals to handling. Corticosterone is the primary glucocorticoid produced and secreted by the adrenal cortex in mice. It is often referred to as the “stress hormone” as it is involved in the stress response and affects blood pressure, blood sugar levels and other actions of stress adaptation [13,27]. The sampling procedure itself can be a source of stress, and it would be ideal if one could measure it with non-invasive techniques. In this way, faecal sampling must be considered a valid non-invasive method for steroid hormone assessment in laboratory mice and rats [14,25,28,29]. 

This study aims to compare three different blood sampling methods (saphenous vein phlebotomy, caudal vein phlebotomy and tail cut blood collection) and the influence of acclimation to the handling needed in these sampling methods to assess which blood sampling method is less stressful and whether stress can be reduced by acclimation techniques, together with the haematological and faecal detection of “stress markers” (glucose and corticosterone faecal metabolites (FCMs)), with the additional focus on sex. The experiment was performed with C57Bl/6J mice, one of the most widely used inbred strain of mice in biomedical research and a background strain to most genetically modified mouse models [30]. Considering different degrees of handling of the three blood sampling methods, differences in “stress markers” (glucose levels and FCMs) were expected [10,28].

## 2. Materials and Methods

### 2.1. Ethical Statement

All procedures were previously approved by the Ethics Committee of the Principe Felipe Research Center according to the National Law for the Protection of laboratory animals (RD 53/2013) and the European Union (European Directive 2010/63/EU)**.**

### 2.2. Animals and Housing Conditions

Twenty-four SPF (specific pathogen-free) young adult male and female C57Bl/6J mice at the age of 10 to 12 weeks were studied. Mice were obtained from a commercial supplier (Charles River Laboratories, France) and randomly allocated to the different groups. They were housed in SPF conditions according to the FELASA guidelines in pressurised and individually ventilated 1145T (403 × 165 × 174 mm; 435 cm^2^ floor area; Tecniplast) cages (70 air changes/h) with irradiated feeding (2014, Envigo, Barcelona, Spain) and autoclaved water. Nesting material and an autoclaved cardboard cylinder were used. Animals were allowed to acclimatise for ten days before experimentation. The light/dark cycle in the animal room consisted of a 12 h/12 h cycle. The temperature was 21 ± 2 °C, with a relative humidity of 50 ± 5% and 15 complete changes of filtered air per hour. Mice were housed in groups of 4 animals to minimise the impact of individual housing. During the sample collection, animals were housed in the same described conditions and all manipulations and sample collections were obtained in diurnal rhythms, between 10:00 and 12:00 in the morning. 

### 2.3. Experimental Design

**Figure 1:**
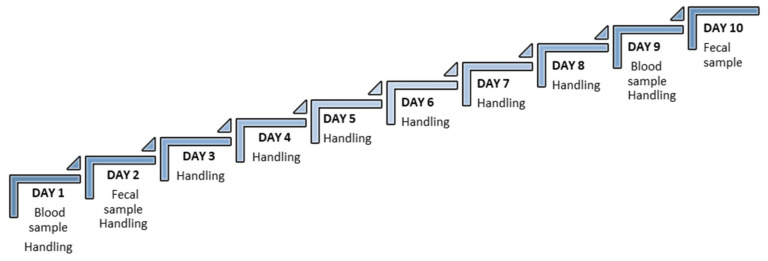
Experimental design timetable. Blood sampling was performed on day 1 and 9. Faecal sampling was performed on day 2 and 10.

Mice were randomly housed, separated by sex and in groups of 4 mice per cage. Cages were randomly divided into the 3 experimental groups: tail vein (TV) group, saphenous vein (SV) group and tail cut (TC) group. Blood samples were obtained before and after acclimation: (a) non-acclimation (PRE-acclimation), where measures were taken without a handling routine; and (b) with acclimation (POST-acclimation), where the animals were trained with the habitual handling needed for blood sampling, as described below (Figure 1). 

### 2.4. Handling Technique

Handling acclimation was always performed in an adjacent experimental room and the mice were transported in their home cage. 

A manipulation routine was established based on the handling needs for each of the three blood sampling methods of the study. Therefore, a specific handling technique was developed for each group, with a duration of 60 s per day for 9 continuous days. Procedures were performed by the same two trained and experienced technicians. 

Based on other studies [10,11,12], the technique has the next sequence:

1. First, animals were placed between the hands of the technician, (as a cave) for about 30 s.
2. Then, the necessary immobilisation for each blood sampling method was performed for another 30 s: the animals from the TC and TV groups were placed on the cage rack and their tails were held, simulating the hold necessary for the blood sampling method, for 30 sec. The animals from the SV group were introduced to a containment “tunnel/tube”, used later as a trap for sampling for 30 s [14,15].

### 2.5. Blood and Faecal Sampling

Blood and faecal sampling were always performed in an adjacent experimental room and the mice were transported in their home cage. 

Glucose levels were obtained using a glucometer Contour^®^XT (Bayer AG, Leverkusen, Germany) according to the manufacturer’s instructions, with a range of detection of 10 mg/dL to 600 mg/dL of glucose.

After blood sampling, mice were transferred to clean cages and faeces were collected 24 h after in all groups, following the protocol described by DetectX^®^. Then, 200 µgr of dried faecal samples were collected into a tube and stored at −21 °C until analysis, as the protocol described. Studies in male and female C53Bl/6 mice report the FCM peak radioactivity to be about 10 h (range 8–12 h) after injection [16]. In addition, corticosteroid hormone secretion is usually pulsatile and influenced by feed intake and environmental factors [17]. In this sense, collection samples from the cage 24 h after blood sampling include the FCM peak, which avoids the problems of circadian variation and timing of metabolism and excretion and is a good method for both chronic and acute studies [16].

Samples were identified by condition, not individually, to avoid the need to individualise animals and minimise this source of stress [31]. The use of this kind of sample that does not require restraining animals is a good method to avoid the effect of hormone secretion. The FCMs were extracted and quantified by enzyme immunometric assay (EIA) according to the manufacturer’s instructions using a DetectX^®^ Cortisol Immunoassay kit, with a sensitivity of 27.6 pg/mL and a detection limit of 45.4 pg/mL.

#### 2.5.1. Tail Vein (TV)

The lateral caudal vein was pricked and a 25-gauge needle was used to puncture the vein. A blood drop was applied on to the glucose strip. Bleeding was stopped by applying a slight pressure with fingers.

#### 2.5.2. Saphenous Vein (SV)

The sample was obtained using a similar technique described by Hem and cols. [18]. The mice were restrained, and introduced into a 20 mL falcon. This restraint allowed them to breathe through a falcon pipe hole at the top. The hind leg was externalized to visualise the saphenous vein. Hair over the saphenous vein was shaved and a 25-gauge needle was used to puncture the vein. After the sample was obtained, slight pressure was applied to stop any bleeding. 

#### 2.5.3. Tail Cut (TC)

To obtain samples via cutting the tail, mice were located on the rack cage, allowing movement as tail vein groups, and a surgical scalpel was used to obtain the sample. In the cut zone, haemostatic powder (Bioline Pet Styptic Powder) was applied to stop the bleeding that habitually cannot be stopped with finger pressure alone. 

### 2.6. Statistics

The sample size was calculated and recognised as statistically significant if a minimum difference of 20 units between any pair of groups in the 3 groups existed, accepting an alpha risk of 0.05, and a beta risk of 0.2 in a two-sided test. In this condition, a sample size of four animals per group was established. 

Data were summarised using mean (standard deviation) and median (1st–3rd quartile) in the case of continuous variables, and by absolute frequencies in the case of categorical variables. The Shapiro–Wilk test confirmed the normal distribution of levels of glucose in all groups. Comparisons between different groups were made using the *t*-test (2 groups) and one-way ANOVA, followed by multiple comparisons (more than two groups). P values lower than 0.05 were considered statistically significant. All analyses were performed using R (version 3.5.2, Foundation for Statistical Computing, Vienna, Austria).

## 3. Results

### 3.1. Differences between Sexes

**Figure 2:**
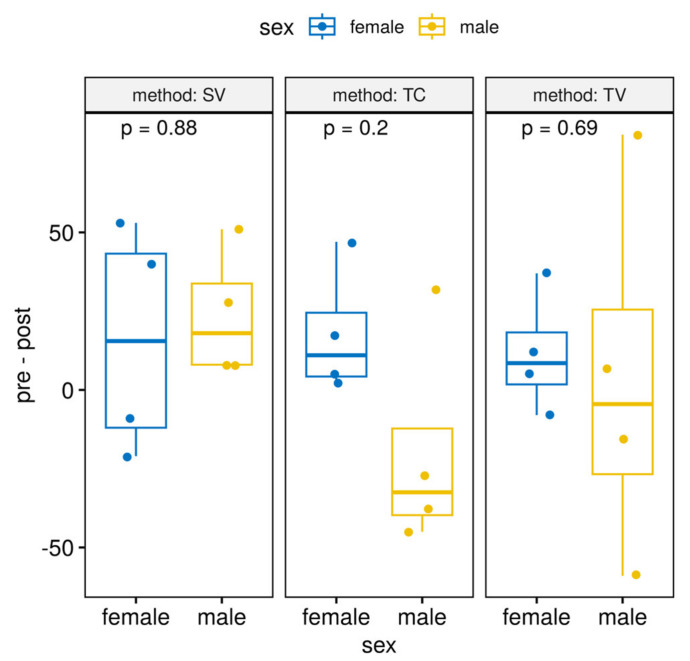
Differences between glucose levels pre and postacclimation between sexes. Results showed no differences by sex in all methods used for sampling (TC group *p* = 0.2; TV group *p* = 0.69; SV group *p* = 0.88).

Results obtained analysing FCM levels (Table 1) and glucose levels (Figure 2) show there were no differences between sexes.

This is interesting because it is not necessary to use a specific method by sex, and this allows us to analyse all animals by technique, without taking into account this variable, the sex. It also allows us to assess the set of all animals as a single group (instead of separate males and females), so we have a higher sample size for the analysis strategy.

### 3.2. Differences between Acclimation and Non-Acclimation

**Figure 3:**
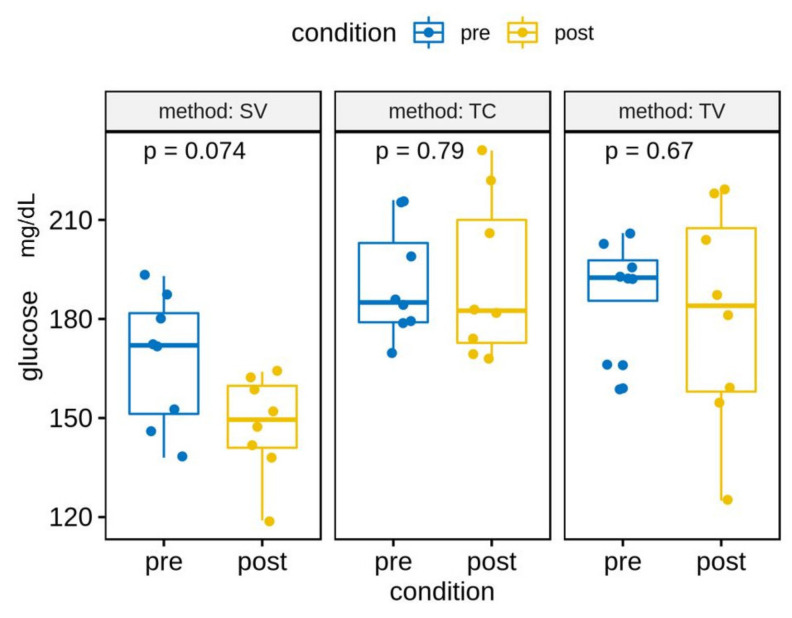
Glucose levels (mg/dL) by technique before (pre-) and after (post-) acclimation.

Overall, the mean glucose levels were lower in POST- than PRE-acclimation, but these differences were not statistically significant. We see the same trend when analysing the glucose levels by technique (Figure 3).

### 3.3. Differences between Measurement Methods

**Figure 4:**
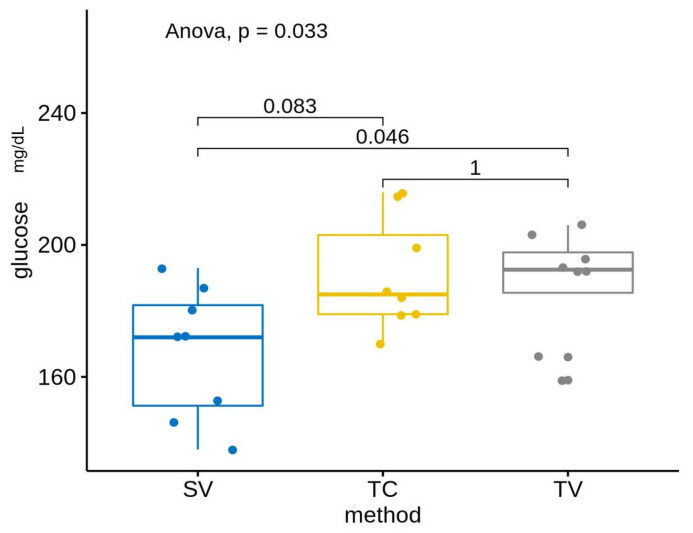
Differences of glucose levels between methods, without acclimation. Results show differences were statistically significant between the SV and TC groups (*p* = 0.083), and between the SV and TV groups (*p* = 0.046).

**Figure 5:**
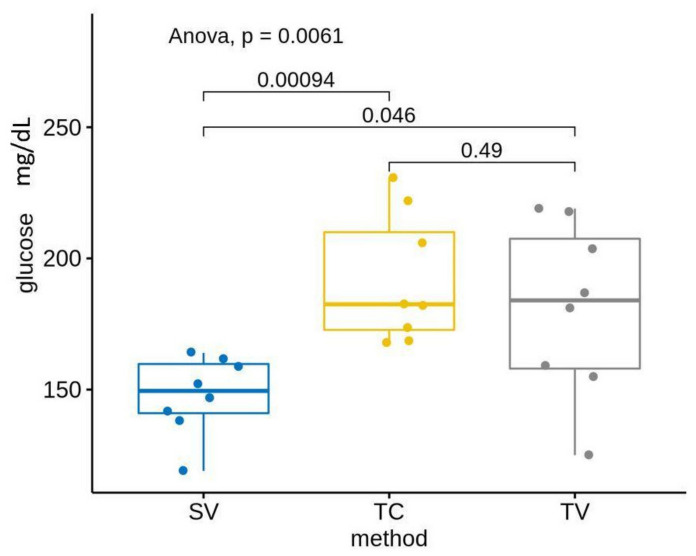
Differences of glucose levels between methods, post-acclimation period. Results show differences were statistically significant between the SV and TC groups (*p* = 0.00094) and between the SV and TV groups (*p* = 0.046).

Our results show that the mean glucose levels differed in the three evaluated methods: SV, TC and TV before and after acclimation (Table 2, Figure 4 and Figure 5). Specifically, glucose levels were lower in the SV group compared with the other two methods: TV and TC (both in PRE as in POST). These results showed statistically significant differences without acclimation between groups: SF–TC (*p*-value < 0.05) and SV–TV (*p*-value < 0.1). Statistically significant differences were shown post-acclimation between groups: SV–TC (*p*-value < 0.05) and SV–TV (*p*-value < 0.05).

### 3.4. Faecal Corticosterone Metabolites Levels

**Figure 6:**
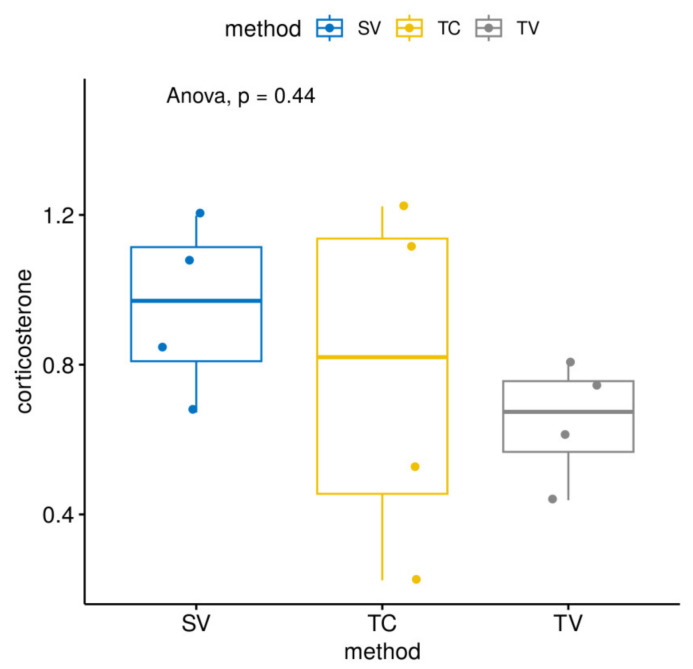
Differences in FCMs (pg/mL) comparing by methods (*p* = 0.44).

Regarding FCM levels by method, we saw lower FCM levels in the TC groups without these differences being significant (Figure 6). 

**Figure 7:**
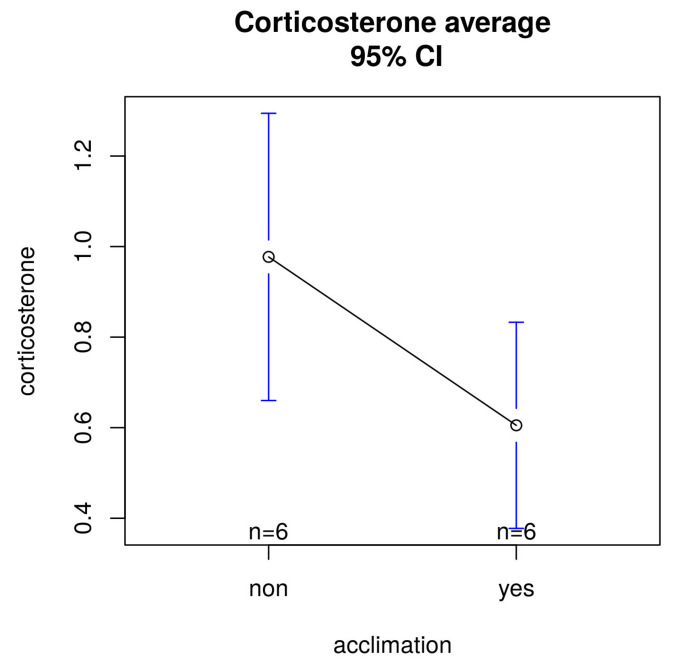
Differences in FCMs (pg/mL) comparing groups with (mean: 0.6052 pg/mL) and without acclimation (mean: 0.9772 pg/mL).

Comparing groups with or without acclimation, FCM levels were significantly higher in measurements without acclimation compared with those with acclimation (Figure 7) but without statistical significance (*p* = 0.078).

## 4. Discussion 

The study reported here compares the impact of widely used blood sampling methods on glucose and FCM levels in C57Bl/6J mice. Plasma glucose in blood samples obtained from the tail and saphenous vein by different methods was measured. Faecal corticosterone was measured by obtaining faeces directly from cages without any kind of manipulation. In mice, the use of caudal veins in different methods or the use of saphenous phlebotomy are common techniques for blood sampling [17,18,20,21]. Some studies evaluate and compare some of them, analysing the quality of blood samples or how stressful the use of such techniques is for animals [17,26,32]. However, the handling need for these techniques is not considered and it is well known that handling is a source of stress for animals [12,22]. When mice are handled using a home cage or external tunnel, they show less anxiety in an elevated plus maze than those picked up by the tail [9,24]. Further, compared with mice picked up by the tail, mice handled by non-aversive tunnel or cupping methods have reduced plasma corticosterone, reduced blood glucose and improved glucose tolerance [33]. Monitoring endocrine functions in mice is constrained seriously by the adverse effects of blood sampling [34,35]. Therefore, non-invasive techniques to monitor stress hormones are highly demanded in the laboratory as well as in field research [10,29]. 

Our results show lower glucose levels when saphenous phlebotomy is used comparing groups with or without acclimation (mean PRE: 167.6 mg/dL; mean POST: 147.6 mg/dL). These results are not statistically significant but show an important reduction close to significance. Routine acclimation was obtained based on different studies [9,22]. It has been described that prior manipulation and habituation reduce anxiety and stress in mice, facilitate routine management, improve animal welfare, decrease shortages of data and improve experimental reliability [10]. However, there is no standard and established technique so we have to consider that these results could be due to a short routine acclimation period, and it would be interesting to consider longer periods when blood sampling techniques are needed.

Groups in which the tail needed to be manipulated did not show this downward trend. Despite the beneficial effects of handling being known, the tail-pick-up approach, which is particularly stressful, is still widely used. Some handling procedures such as picking up animals by the tail may actually simulate the act of being captured and provoke stress responses [9,36]. The method in which a saphenous vein was used for sampling (SV) resulted in lower glucose levels. This technique, which apparently needs high manipulation and could be more stressful than TC or TV, without less handling, could be considered as a less stressful method. These results confirm that tail manipulation is a stressful technique, as other studies have shown [32,37,38]. In recent years, less aversive handling methods (for example, tunnel handling or bowl hand) have been shown to mitigate anxiety and depressive behaviours in mice [24,39]. In this sense, the SV sampling method allows the animals to be sheltered (hidden) in the tunnel while the sample is taken, while, in the other two methods, the animals cannot hide, and the tail is manipulated to a greater or lesser extent. The standard handling method of picking up mice by the tail increases behavioural and physiological measures of stress and anxiety, which may explain our results [24,32,35,40].

Comparing groups by technique without acclimation, we confirmed animals manipulated by the tail (TC and TV groups) showed no differences between them, while the differences between the saphenous group and the TC group were statistically significant (*p* = 0.046). Kress and cols. considered the saphenous technique more stressful than the puncture of the facial vein because of the time required for sampling and the increase of corticosterone in urine production, but our results show lower glucose levels, indicating that the SV method is less stressful than the TV or TC methods [20]. Comparing techniques after the handling routine, after the acclimation period was completed, we saw similar results in which the statistically significant differences were between the SV and TV groups, with a *p*-value = 0.046, and between the SV and TC groups, with a *p*-value = 0.00094. These results confirm that manipulation of the tail and the handling needed for a tail vein phlebotomy or a tail vein docking is a stressful technique, with the independence of a previous acclimation period. 

As an additional measure of stress levels, faecal samples were collected to measure FCMs. Typically, blood serum or plasma is used to measure corticosterone concentrations, with an increase implying an acute stress response [27], but the measurement of FCMs has been proposed due to the advantage of it being a non-invasive technique. Corticosterone in the blood can usually be demonstrated after a few minutes but it requires a quick analysis because of its short half-life in plasma [11,19]. Nevertheless, corticosterone is a stable metabolite to detect in faeces. In fact, measurement of FCMs has become a common approach in evaluating HPA activity because it is non-invasive and provides a relatively more stable and time-integrated picture of HPA activation than do circulating corticosterone levels [41]. Therefore, in order to avoid the activation of the HPA axis, which quickly leads to the secretion of glucocorticoids associated with restraint and manipulation needs to blood sampling, we analysed faecal corticosterone metabolites using DetectX^®^ Cortisol Immunoassay, as the protocol described. To minimise interaction with animals to stop them stressing or influence the results we analysed faecal metabolites by cage 24 h after sampling [37]. It has been demonstrated that 24 h FCM collection avoids the problems of circadian variations and it is a good method for both chronic and acute studies [14]. Our results reflected a reduction in FCMs after acclimation but without statistical significance. However, since we only measured the total amount of FCMs excreted 0–24 h after blood sampling by cage, additional faecal sampling would be necessary to assess whether there is an effect of blood sampling on FCMs. 

We know that isolation allows easier collection of faeces and would have increased the number of samples, but it has been demonstrated to induce an increase in corticosterone levels with respect to animals housed in standard cages, in groups of two-to-three per cage [42]. Alterations in neurochemistry, metabolism, growth, reproduction and dopaminergic hyperactivity have been found in shared neural regions, implicated with the performance of stereotypy in isolated mice. Animals habituated to stable groups show less stress than when in individual housing [18,43], but our results do not show differences between methods and between pre- and post-acclimation (*p* = 0.078). The TV method shows lower FCM metabolites than the other methods, and FCM levels are significantly higher without acclimation than those obtained with acclimation.

Interestingly, there are some studies concerning the differences in anxiety and stress responsiveness by sex [42,44], but we found no statistically significant differences in this respect. Hurst and West demonstrated similar responses to the different handling methods in males and females mice. Further, their studied ICR and C57Bl6/J strains, and both strains showed the same general differences in responses on tunnel and tail handling [9]. On the basis of the results obtained in this work, the following studies will be proposed in which some methodological limitations concerning the experimental design or the consideration of multifactorial designs in the analysis will be improved, allowing joint treatment of all the variables of interest, with a global approach to all the possible sources of variability. In light of all these data, we suggest the use of the SV technique for blood sampling as a less stressful method and highlight the importance of acclimation in any manipulation and any technique in which restraint is needed. 

## 5. Conclusions 

We conclude that acclimation must be considered a requirement to minimise stress in mice blood sampling techniques. The use of the saphenous vein for blood sampling, despite the handling needed, could be considered a less stressful technique than tail docking or tail phlebotomy. 

These results allow researchers to select the most welfare-friendly blood sampling technique objectively from studied techniques for a given experiment and contribute to refinement within the 3R concept, the essential concept on which laboratory animal science is based.

## Figures and Tables

**Table 1:** FCM levels (pg/mL) description by acclimation (PRE, POST) and sex (Female, Male). Descriptives: sample size, minimum, 1st quartile, median, mean, 3rd quartile, maximum, standard deviation, t-statistic, *p* value.

PRE	n	Min.	1st Qu.	Median	Mean	3rd Qu.	Max.	t	*p* Value
Female	12	0.438	0.762	1.086	0.916	1.155	1.223	0.457	0.680
Male	12	0.810	0.959	1.108	1.039	1.153	1.198		
**POST**	**n**	**Min.**	**1st Qu.**	**Median**	**Mean**	**3rd Qu.**	**Max.**	**t**	***p* Value**
Female	12	0.532	0.635	0.738	0.708	0.797	0.855	1.221	0.298
Male	12	0.224	0.417	0.610	0.502	0.641	0.672		

**Table 2:** Description of glucose levels (mg/dL) by acclimation and sampling methods. Descriptives: sample size, minimum, 1st quartile, median, mean, 3rd quartile, maximum, standard deviation.

		n	Min.	Q1	Median	Mean	Q3	Max.	SD
PRE	TC	8	170	179	185	191	203	216	17.2
	TV	8	159	185.5	192.5	188.4	197.8	206	16.9
	SV	8	138	151.2	172	167.6	181.8	193	19.9
POST	TC	8	168	172.8	182.5	191.9	210	231	24.6
	TV	8	125	158	184	181	207.5	219	33.1
	SV	8	119	141	149.5	147.9	159.8	164	15

## Data Availability

All the data of the study can be made available upon request.
